# Sport-related differences in QT dispersion and echocardiographic parameters in male athletes

**DOI:** 10.1038/s41598-023-33957-8

**Published:** 2023-04-25

**Authors:** Viktor Stoičkov, Dragan Radovanović, Marina Deljanin-Ilić, Zoran Perišić, Milan Pavlović, Ivan Tasić, Ivan Stoičkov, Mlađan Golubović, Aaron T. Scanlan, Vladimir Jakovljević, Emilija Stojanović

**Affiliations:** 1 Institute for Treatment and Rehabilitation “Niska Banja”, Clinic for Cardiovascular Diseases, Niš, Serbia; 2 Faculty of Medicine, Department of Internal Medicine, University of Niš, Niš, Serbia; 3 Faculty of Sport and Physical Education, University of Niš, Niš, Serbia; 4 Cardiovascular Diseases Clinic, Clinical Center Niš, Niš, Serbia; 5 Human Polyclinic, Niš, Serbia; 6 Cardiovascular and Transplant Surgery Clinic, Clinical Center Niš, Niš, Serbia; 7 School of Health, Medical and Applied Sciences, Central Queensland University, Rockhampton, Australia; 8 Department of Physiology, Faculty of Medical Sciences, University of Kragujevac, Kragujevac, Serbia; 9 Department of Human Pathology, Moscow State Medical University IM Sechenov, Moscow, Russia

**Keywords:** Cardiology, Health care, Medical research

## Abstract

The aim of this study was to compare QT dispersion (QTd) and echocardiographic parameters in male athletes competing across different sports (long-distance running, volleyball, football, powerlifting, and bodybuilding) and a control population. Significant *moderate-strong* differences (p < 0.001, ${\eta }_{p}^{2}$ = 0.52–0.71) were found in corrected QTd, intraventricular septal wall thickness (ISWT), posterior wall thickness (PWT), relative wall thickness (RWT) and LV (left ventricular) index between groups. Corrected QTd, ISWT, PWT, and RWT were significantly (p < 0.001) higher in powerlifters and bodybuilders compared to other athlete groups and controls. While all athlete groups displayed a significantly higher LV index (p < 0.05) compared to controls, corrected QTd was significantly lower (p < 0.001) only in long-distance runners, volleyball athletes, and football athletes compared to controls. Normal or eccentric LV hypertrophy (LVH) was observed in most long-distance runners (58% and 33%), volleyball athletes (50% and 50%), and football athletes (56% and 41%). In contrast, concentric LVH was observed in most powerlifters (58%) and bodybuilders (54%). Advanced LVH, predominantly concentric in nature, appears to be accompanied with increased QTd in powerlifters and bodybuilders. On the other hand, runners, volleyball athletes, and football athletes experienced LVH toward the upper threshold of the normal reference range alongside reduced QTd compared to other groups.

## Introduction

Prolonged QT dispersion (QTd) derived from electrocardiograms has been used as an indicator of abnormal ventricular repolarization for several cardiac diseases^1^. Many studies suggest that increased QTd is associated with increased risk of ventricular arrhythmias^1–3^ and sudden death^4–6^. In this regard, an increased QTd has been documented in patients with arterial hypertension and left ventricular (LV) hypertrophy^7^. In addition to clinical environments, LV hypertrophy is observed in athletes completing regular training^8–10^. The LV hypertrophic cardiac response serves as a reactive mechanism to compensate for volume overload experienced during endurance training or pressure overload experienced during resistance training^11,12^. Although athletes develop impressive LV tissue growth, it is unknown whether this exercise-induced LV hypertrophy is associated with increased QTd like that observed in patients with hypertensive myocardial hypertrophy^7^.

Increased interest has emerged in quantifying QTd, either in isolation^13,14^ or combined with echocardiographic parameters^15–20^ in athletes and the general population. While the available evidence suggests that exercise may affect echocardiographic parameters (including increased LV-end diastolic diameter^16,18–20^, LV end-systolic diameter^16^, LV-end diastolic and systolic volume^16^, LV mass^17,19,20^, posterior wall thickness^19,20^, septal wall thickness^20^, and LV ejection fraction^20^) in athletes, research directly comparing between athletes and sedentary control groups has yielded equivocal findings pertaining to QTd^16–20^. Namely, similar,^13,14,18,19^ lower,^17^ or even higher^16,20^ corrected QTd measures have been reported in athletes compared to control groups. Inconsistent findings across studies potentially reflect disparities in QTd across sexes (male^13,14,16,18–20^ and female^13,16,20^), age groups (adolescent^16^, adult^13,14,17,19,20^, and middle-aged subjects^18^), and sports (combat sports^13,18^, team ball sports^13,16,18^, racket sports^13,18^, track and field^13,14,18–20^, cycling^13^, swimming^18^, and gymnastics^18^).

Despite evidence suggesting that cardiac remodeling varies in response to pressure overload (concentric hypertrophy) or volume overload (eccentric hypertrophy)^10,21^, limited research has compared QTd and echocardiographic parameters across athletes competing in different sports varying in terms of the predominant type of overload imposed. In this way, sports may be classified according to the mechanical demands they elicit^22^ based on the peak static components (expressed as relative intensity of voluntary muscular contraction across three levels [A, B, C]) and the peak dynamic components (expressed as percentage of maximal oxygen uptake across three levels [I, II, III]) undertaken by athletes. To our knowledge, only three studies have compared electrocardiographic parameters in isolation^14^ or combined with echocardiographic parameters^15,19^ across athletes competing in sports with different mechanical demands, showing similar^15^ or prolonged^14,19^ QTd in athletes undertaking static training compared to athletes undertaking dynamic training as their predominant form of exercise. The equivocal findings across studies directly comparing QTd between athletes predominantly undertaking static and dynamic forms of exercise or between athletes and control groups suggest more research is needed to develop a definitive consensus regarding the sport-specific cardiac features developed in athletes^23^. Also, no evidence is available concerning the associations between QTd and echocardiographic parameters in athletes. In addition to indicating sport-specific cardiac features, QTd and echocardiographic data in athletes will provide insight as to whether structural cardiac remodeling in response to training produces abnormalities in cardiac function and QTd, which is vital in distinguishing an athletic heart from pathological cardiac hypertrophy. Therefore, the aims of this study were to: 1) compare QTd and echocardiographic parameters between athletes competing in different sports and sedentary controls; and 2) assess the associations between QTd and echocardiographic parameters among these athletes.

## Methods

### Participants

A total of 130 highly trained male athletes competing at the national level in their respective sport and 43 age-matched healthy controls with a sedentary lifestyle volunteered to participate in this study. Athletes consisted of long-distance runners (n = 12), volleyball athletes (n = 14), football athletes (n = 39), powerlifters (n = 26), and bodybuilders (n = 39). Analyses using G*power software (version 3.1.9.4; Heinrich Heine University Düsseldorf, Düsseldorf, Germany) indicated our study was sufficiently powered given n = 150 was recommended [alpha = 0.05, effect size = 0.30; power = 0.80], based on research examining the influence of sex and type of sport on QTd in elite athletes^14^. Inclusion criteria for participation in the study were as follows: (a) at least 3 years of training and competition experience immediately prior to participation in the study, performing ≥ 6 h·week^−1^ of exercise (for athletes); (b) compliance with the banned substance list; (c) free from injury/illness; (d) 18–39 years of age; and (e) sedentary participants to have never been involved in any sports training or competition and completing < 2 h·week^−1^ of physical exercise prior to the study. The exclusion criteria were as follows: (a) history of cardiovascular disorders; (b) history of diabetes mellitus; (c) history of arterial hypertension; (d) history of dyslipidemia; or (e) use of medications known to alter cardiac conduction. Training characteristics (frequency, duration, and type) for the athletes recruited in this study are described in Table 1. This study was approved by the Faculty of Medicine Research Ethics Committee with all procedures conducted in accordance with the Helsinki Declaration. All participants provided informed consent prior to participation, approved by the Faculty of Medicine Research Ethics Committee.

**Table 1:** Percentage (%) of the total daily training time devoted to general physical conditioning, specific physical conditioning, and/or technical-tactical skills training in each athlete group.

Training characteristics	Monday	Tuesday	Wednesday	Thursday	Friday	Saturday	Sunday
**Long-distance runners** (6 × 60–90-min sessions per week)	70–110 km per week at speeds of 8–10 km h^−1^					Competition or training	Day off
Physical conditioning							
**Volleyball athletes** (6 × 90-min sessions per week)						Game	Day off
General physical conditioning (%)	30	20	10	10	10		
Specific physical conditioning (%)	30	30	50	40	40		
Technical-tactical skills training (%)	40	50	40	50	50		
**Football athletes** (6 × 90–120-min sessions per week)						Game	Day off
General physical conditioning (%)	40	30	20	10	10		
Specific physical conditioning (%)	40	40	30	40	40		
Technical-tactical skills training (%)	20	30	50	50	50		
**Powerlifters** (6–10 × 90-min sessions per week)						Competition or training	Day off
General physical conditioning (%)	15	15	15	10	10	10	
Specific physical conditioning (%)	85	85	85	90	90	90	
**Bodybuilders **(6–10 × 90-min sessions per week)						Competition or training	Day off
General physical conditioning (%)	50	30	30	20	10	10	
Specific physical conditioning (%)	50	70	70	80	90	90	

General physical conditioning in volleyball and football athletes included continuous running, short sprints, plyometric jumps, upper-extremity exercises, and trunk and core stability exercises; Specific physical conditioning in volleyball and football athletes included jumps and sprints combined with technical exercises aimed at improving sprinting speed and agility with and without the use of balls; General physical conditioning in powerlifters included jogging on a treadmill at 4–6 km·h^−1^; Specific physical conditioning in powerlifters (60–95% of 1 repetition maximum [1RM], ≤ 10 repetitions) and bodybuilders (60–80% of 1RM, 12–20 repetitions) consisted of bench press, lateral pull-down, standing shoulder press, arm curl and extension, leg press, squat, leg curl, knee extension, calf press, abdominal crunch, and dead lift exercises; General physical conditioning in bodybuilders included jogging on a treadmill at 4–7 km·h^−1^ and high-intensity interval training (1 session per week).

### Procedures

A cross-sectional experimental design was adopted. All participants underwent clinical examination including a medical interview, measurement of general characteristics (age, height, body mass index [BMI], and body surface area), as well as electrocardiographic and echocardiographic screening at the Clinic for Cardiovascular Diseases. All assessments were carried out in the morning between 08:00 and 10:00.

### Electrocardiography

A 12-lead electrocardiogram was recorded after a 5-min resting period in the supine position using a conventional surface electrocardiogram device (Schiller AT-10 plus; Schiller AG; Baar, Switzerland) at 25 mm/s and 1 mV/cm voltage. The QT interval was measured manually from the onset of the QRS complex to the end of the T wave, defined as the point of return of the T wave to the isoelectric line or to the nadir between the T and U waves in cases where a U wave was present. If the end of the T wave could not be determined reliably, or when the T wave was isoelectric or of very low amplitude, the QT measurement was not made in that lead and was excluded from the analysis. In all participants, the QT interval was measurable in at least nine electrocardiogram leads. QTd was calculated as the difference between the longest and shortest QT interval. Corrected QTd was defined as the difference between maximum and minimum corrected QT (QTc) interval. QT interval was corrected for heart rate by using Bazett’s formula [QTc = QT/√(R–R interval)]. R–R interval was determined as: 60/ heart rate. All electrocardiograms were read by two experienced cardiologists, with any disagreements resolved through discussion, or consulted with a third cardiologist for a consensus decision. The intra- and inter-rater reliability (coefficient of variation percentage) for QT measurements were 5% and 10% respectively.

### Echocardiography

Two-dimensional M-mode echocardiograms were performed using commercially-available equipment (Siemens Acuson SC2000; Siemens Medical Solutions USA, Inc.; Mountain View, CA, USA). All measurements were performed by the same experienced cardiologist in accordance with the guidelines of the American Society of Echocardiography^24,25^. Intra-rater variability (coefficient of variation percentage assessed from a random sample of 10 echocardiographic measurements) was < 5%. A second cardiologist was available and consulted for opinion or re-measurement in cases where there was any uncertainty. Left atrium diameter, aortic root diameter, LV end-diastolic diameter, LV end-systolic diameter, intra-ventricular septal wall thickness (ISWT), posterior wall thickness (PWT), and LV mass were taken in the parasternal long-axis view. LV mass and aortic root diameter were indexed to body surface area. Body surface area (BSA) was determined as^26^: BSA = 0.007184 * height^0.725^ * body mass^0.425^. Relative wall thickness (RWT) was calculated as: (2 × PWT)/LV end-diastolic diameter. LV geometric pattern was characterized as either^24^: (1) concentric hypertrophy (LV index > 115 g/m^2^, RWT > 0.42); (2) eccentric hypertrophy (LV index > 115 g/m^2^, RWT < 0.42); (3) concentric LV remodeling (LV index < 115 g/m^2^, RWT > 0.42); or a (4) normal pattern (LV index < 115 g/m^2^, RWT < 0.42).

Transmitral diastolic blood flow was registered using pulsed-wave Doppler from the apical position of the transducer using the section of the four-chamber view. The sample volume was placed at the level of the top of the mitral leaflets in the open position. Diastolic transmitral flow velocities (early [E] and late [A]), E/A ratio, and deceleration time of the LV were determined. Systolic function of the LV was determined using LV ejection fraction (automatically derived from end-diastolic volume and end-systolic volume) and fractional shortening. The peak systolic (s′), as well as early (e′) and late (a′) diastolic mitral tissue velocities were measured at the medial and lateral side of the mitral annulus. E/e′ was also calculated.

### Statistical analysis

Data were analyzed using IBM SPSS Statistics (v24.0; IBM Corporation, Armonk, NY, USA), with statistical significance accepted at p ≤ 0.05. Normality of all data was verified with quantile–quantile (Q-Q) plots, as well as skewness and kurtosis coefficients. All data are expressed as the mean ± standard deviation. Differences in general characteristics, electrocardiographic, and echocardiographic variables between long-distance runners, volleyball athletes, football athletes, powerlifters, bodybuilders, and sedentary controls were assessed using one-way analyses of variance (ANOVA) with Bonferoni post-hoc tests. The effect size of each ANOVA was determined using partial eta squared (${\eta }_{p}^{2}$) and interpreted as: *no effect* (≤ 0.03), *minimum effect* (0.04–0.24), *moderate effect* (0.25–0.63), and *strong effect* (≥ 0.64). Associations between corrected QTd and echocardiographic parameters were determined using Pearson product-moment correlations with 95% confidence intervals (CIs). Correlation magnitudes were interpreted as^27^: *trivial* (0–0.10), *small* (0.11–0.30), *moderate* (0.31–0.50), *large* (0.51–0.70), *very large* (0.71–0.90), *almost perfect* (0.91–0.99), and *perfect* (1.00).

## Results

General characteristics and electrocardiographic parameters for long-distance runners, volleyball athletes, football athletes, powerlifters, bodybuilders, and sedentary controls are presented in Table 2.

**Table 2:** Demographic variables (mean ± standard deviation) and electrocardiographic parameters in long-distance runners (n = 12), volleyball athletes (n = 14), football athletes (n = 39), powerlifters (n = 26), bodybuilders (n = 39), and sedentary controls (n = 43).

Outcome measure	Long-distance runners	Volleyball athletes	Football athletes	Power lifters	Bodybuilders	Sedentary controls	ANOVA		
							p		${\eta }_{p}^{2}$
Demographic variables									
Age (years)	30.17 ± 6.59	25.79 ± 5.20	27.54 ± 6.13	27.92 ± 6.04	28.31 ± 6.46	28.47 ± 5.60	0.548	0.024	
Body height (cm)	181.25 ± 6.28*	193.93 ± 8.74^║^	181.69 ± 6.31*	181.15 ± 6.84*	179.89 ± 7.14*	182.93 ± 5.29	** < 0.001**	0.233	
Body surface area	1.99 ± 0.13*	2.17 ± 0.17	1.99 ± 0.12*	2.06 ± 0.13	2.07 ± 0.15	2.06 ± 0.16	**0.003**	0.100	
Body mass index	23.93 ± 2.15^#^	22.79 ± 2.59^║^	23.87 ± 1.82^#¶^	26.05 ± 2.45*	27.05 ± 3.06*	25.34 ± 3.14	** < 0.001**	0.214	
Electrocardiographic parameters									
R–R interval (ms)	0.88 ± 0.07	0.91 ± 0.10^║^	0.97 ± 0.13^║#^	0.90 ± 0.13^║^	0.84 ± 0.12	0.77 ± 0.11	** < 0.001**	**0.270**	
QTd (ms)	29.58 ± 6.56^║#¶^	35.00 ± 5.54^║#¶^	33.33 ± 7.46^║#¶^	56.92 ± 8.00^║^	52.18 ± 6.96^║^	42.21 ± 8.47	** < 0.001**	**0.614**	
Corrected QTd (ms)	31.43 ± 6.56^║#¶^	36.73 ± 5.68^║#¶^	34.03 ± 6.66^║#¶^	60.26 ± 7.30^║^	57.41 ± 5.69^║^	47.96 ± 8.20	** < 0.001**	**0.713**	

Bolded p values indicate statistically significant difference (p < 0.05), bolded ${\eta }_{p}^{2}$ indicate moderate to strong effects; body surface area = 0.007184 * height^0.725^ * body mass^0.425^.

*QTd* QT dispersion.

*Significantly different from volleyball athletes.

^#^Significantly different from bodybuilders.

^¶^Significantly different from powerlifters.

^║^Significantly different from sedentary controls.

### General characteristics

Body height, BSA, BMI, and body mass were significantly (p < 0.001–0.003; ${\eta }_{p}^{2}$ = 0.10–0.23, *minimum*) different between groups. Post-hoc analyses revealed a significantly (p < 0.001) higher body height in volleyball athletes compared to all other groups. Also, BSA was significantly higher in volleyball athletes compared to long-distance runners (p = 0.031) and football athletes (p = 0.003). On the other hand, volleyball athletes possessed a significantly lower BMI compared to powerlifters (p = 0.005), bodybuilders (p < 0.001), and sedentary controls (p = 0.008). BMI was also significantly higher in bodybuilders (p < 0.001) and powerlifters (p = 0.022) compared to football athletes, as well as in bodybuilders compared to long-distance runners (p = 0.008). Body mass was significantly higher in bodybuilders compared to football athletes (p = 0.003).

### Electrocardiographic parameters

RR-interval, QTd, and corrected QTd were significantly (p < 0.001; ${\eta }_{p}^{2}$= 0.27–0.71, *moderate-strong*) different between groups. Post-hoc analyses revealed a significantly shorter RR-interval in sedentary controls compared to volleyball athletes (p = 0.004), football athletes (p < 0.001) and powerlifters (p = 0.001). Also, the RR-interval was significantly shorter (p < 0.001) in bodybuilders compared to football athletes. Bodybuilders and powerlifters exhibited a significantly higher QTd and corrected QTd (p < 0.001) compared to all other groups. In addition, sedentary controls exhibited a significantly higher QTd compared to long-distance runners (p < 0.001), volleyball athletes (p = 0.032), and football athletes (p < 0.001). Likewise, corrected QTd was significantly higher in sedentary controls (p < 0.001) compared to long-distance runners, volleyball athletes, and football athletes.

### Echocardiographic parameters

**Figure 1:**
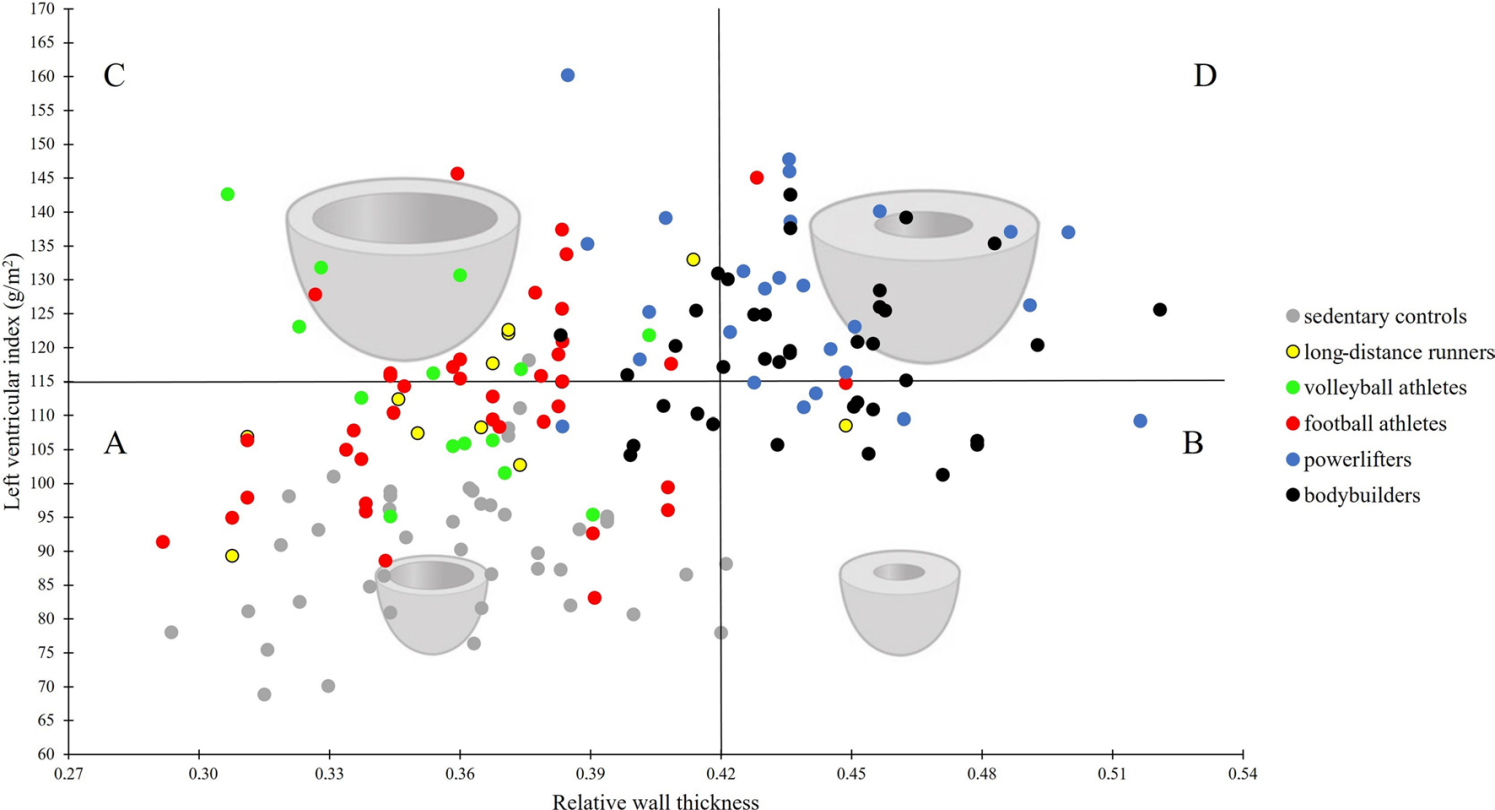
Left ventricular geometric patterns in long-distance runners (n = 43), volleyball athletes (n = 14), football athletes (n = 39), powerlifters (n = 26), and bodybuilders (n = 39) and sedentary controls (n = 43). (**A**) Normal pattern (RWT < 0.42 and LV index < 115 g/m^2^); (**B**) concentric remodeling (RWT > 0.42 and LV index < 115 g/m^2^); (**C**) eccentric hypertrophy (RWT < 0.42 and LV index > 115 g/m^2^); (**D**) concentric hypertrophy (RWT > 0.42 and LV index > 115 g/m^2^).

Echocardiographic parameters for long-distance runners, volleyball athletes, football athletes, powerlifters, bodybuilders, and sedentary controls are presented in Table 3, with correlations between corrected QTd and echocardiographic parameters among these groups presented in Table 4. The LV geometric patterns for each participant in each group are also shown in Fig. 1. Normal or eccentric LV hypertrophy was observed in most long-distance runners (58% and 33%), volleyball athletes (50% and 50%), and football athletes (56% and 41%). To the contrary, concentric LV hypertrophy was observed in most powerlifters (58%) and bodybuilders (54%). Normal geometric pattern was observed in 93% of sedentary controls.

**Table 3:** Echocardiographic parameters (mean ± standard deviation) in long-distance runners (n = 12), volleyball athletes (n = 14), football athletes (n = 39), powerlifters (n = 26), bodybuilders (n = 39), and sedentary controls (n = 43).

Outcome measure	Long-distance runners	Volleyball athletes	Football athletes	Power lifters	Bodybuilders	Sedentary controls	ANOVA	
							**p**	${{\varvec{\eta}}}_{{\varvec{p}}}^{2}$
Structural parameters								
Left atrium diameter (mm)	39.67 ± 2.60^║^	41.77 ± 2.78^║^	40.32 ± 2.84^║^	41.15 ± 3.35^║^	40.19 ± 3.37^║^	37.33 ± 2.12	** < 0.001**	0.216
Aortic root diameter (mm)	32.88 ± 2.13	34.97 ± 1.49^║^	33.45 ± 1.44	33.46 ± 2.14	33.30 ± 2.23	32.76 ± 1.84	**0.011**	0.084
Aortic root index (mm/m^2^)	16.55 ± 1.14	16.20 ± 1.02	16.80 ± 1.11^║^	16.26 ± 0.95	16.12 ± 1.09	15.88 ± 1.03	**0.005**	0.094
LV end-diastolic diameter (mm)	54.82 ± 1.56^║#^	57.30 ± 3.56^║#¶^	55.04 ± 2.78^║#^	53.24 ± 2.87	51.99 ± 2.28	52.08 ± 2.64	** < 0.001**	**0.294**
LV end-systolic diameter (mm)	32.81 ± 1.58	34.25 ± 2.02^#^	33.63 ± 1.87^#^	32.93 ± 2.10	32.21 ± 1.47	32.61 ± 1.90	**0.002**	0.109
Intra-ventricular septal WT (mm)	10.72 ± 0.97^#¶^	11.09 ± 0.77^║#¶^	10.66 ± 0.86^║#¶^	12.53 ± 0.77^║^	12.52 ± 0.93^║^	10.05 ± 0.58	** < 0.001**	**0.631**
Posterior WT (mm)	10.06 ± 0.92^#¶^	10.16 ± 0.50^║#¶^	10.00 ± 0.84^║#¶^	11.63 ± 0.62^║^	11.43 ± 0.61^║^	9.37 ± 0.87	** < 0.001**	**0.587**
Relative WT	0.37 ± 0.04^#¶^	0.36 ± 0.03^#¶^	0.36 ± 0.03^#¶^	0.44 ± 0.03^║^	0.44 ± 0.03^║^	0.36 ± 0.03	** < 0.001**	**0.596**
LV mass (g)	223.12 ± 25.33^║¶^	248.36 ± 36.26^║^	223.43 ± 33.01^║#¶^	262.80 ± 31.31^║^	247.54 ± 29.42^║^	187.52 ± 27.38	** < 0.001**	**0.445**
LV index (g/m^2^)	112.12 ± 11.15^║¶^	114.63 ± 14.19^║¶^	111.86 ± 14.62^║¶^	127.58 ± 13.40^║^	119.53 ± 11.03^║^	90.52 ± 10.54	** < 0.001**	**0.522**
Systolic function								
LV ejection fraction (%)	71.18 ± 2.87^║#¶^	70.07 ± 2.73^║#^	68.94 ± 2.90^║^	68.21 ± 1.81	67.63 ± 1.66	67.25 ± 2.03	** < 0.001**	0.206
Fractional shortening (%)	40.98 ± 2.45^║#¶^	40.20 ± 2.36^║#¶^	39.14 ± 2.45^║^	38.07 ± 2.07	37.88 ± 1.33	37.36 ± 2.08	** < 0.001**	0.221
s'-wave (cm/s)	0.10 ± 0.01	0.10 ± 0.01	0.10 ± 0.01	0.10 ± 0.01	0.11 ± 0.01	0.10 ± 0.01	0.956	0.006
Diastolic function								
Mitral valve inflow peak E (cm/s)	0.92 ± 0.15	0.88 ± 0.15	0.87 ± 0.12	0.94 ± 0.17	0.89 ± 0.14	0.87 ± 0.13	0.338	0.033
Mitral valve inflow peak A (cm/s)	0.52 ± 0.07	0.54 ± 0.12	0.53 ± 0.06^#^	0.58 ± 0.09	0.61 ± 0.13	0.57 ± 0.11	**0.006**	0.093
E/A ratio	1.77 ± 0.33^#^	1.66 ± 0.22	1.67 ± 0.23^#^	1.65 ± 0.26	1.50 ± 0.26	1.53 ± 0.21	**0.002**	0.109
Mitral valve inflow Dt (m/s)	198.92 ± 16.19	207.92 ± 21.03	204.05 ± 19.38	207.15 ± 15.48	209.92 ± 20.40	208.00 ± 17.89	0.507	0.025
eʹ-wave (m/s)	0.17 ± 0.03	0.18 ± 0.03	0.17 ± 0.02	0.17 ± 0.03	0.16 ± 0.02	0.16 ± 0.01	0.104	0.053
aʹ-wave (m/s)	0.10 ± 0.01	0.09 ± 0.02	0.10 ± 0.01	0.10 ± 0.01	0.10 ± 0.01	0.10 ± 0.01	0.204	0.042
eʹ/aʹ	1.74 ± 0.30^║^	2.05 ± 0.51^#^	1.74 ± 0.28*	1.76 ± 0.34	1.67 ± 0.31	1.62 ± 0.25	**0.002**	0.109
E/eʹ	5.52 ± 1.09	4.82 ± 0.54	5.26 ± 0.84	5.63 ± 0.90	5.46 ± 0.83	5.34 ± 0.89	0.093	0.054

Relative wall thickness = (2 × posterior wall thickness)/LV end-diastolic diameter; bolded p values indicate statistically significant difference (p < 0.05), bolded ${\eta }_{p}^{2}$ indicate moderate to strong effects.

*LV* left ventricular, *WT* wall thickness, *Dt* deceleration time, *s'-wave* systolic mitral annulus velocity (average of septal and lateral measurements), *e'-wave* early diastolic myocardial relaxation (average of septal and lateral measurements), *a'-wave* active atrial contraction in late diastole (average of septal and lateral measurements).

*ssignificantly different from volleyball athletes.

^#^Significantly different from bodybuilders.

^¶^Significantly different from powerlifters.

^║^Significantly different from sedentary controls.

**Table 4:** Pearson product-moment correlations between corrected QT dispersion and echocardiographic parameters in long-distance runners (n = 12), volleyball athletes (n = 14), football athletes (n = 39), powerlifters (n = 26), bodybuilders (n = 39), and sedentary controls (n = 43).

Outcome measure	Long-distance runners		Volleyball athletes		Football athletes		Powerlifters		Bodybuilders		Sedentary controls	
	r	p	r	p	r	p	r	p	r	p	r	p
Structural parameters												
Left atrium diameter	0.343	0.275	− 0.033	0.910	0.044	0.789	− 0.023	0.910	0.075	0.651	0.135	0.390
Aortic root diameter	0.464	0.129	0.229	0.431	0.270	0.097	− 0.139	0.498	0.191	0.245	**0.419**	**0.005**
Aortic root index	− 0.022	0.947	− 0.310	0.281	0.018	0.912	− 0.083	0.687	0.106	0.520	− 0.059	0.708
LV end-diastolic diameter	− 0.196	0.542	**0.564**	**0.036**	0.241	0.139	0.064	0.757	0.160	0.332	0.271	0.079
LV end-systolic diameter	− 0.051	0.874	0.482	0.081	**0.409**	**0.010**	0.106	0.605	0.041	0.806	0.241	0.119
Intra-ventricular septal WT	**0.712**	**0.009**	0.292	0.311	**0.517**	**0.001**	**0.559**	**0.003**	**0.406**	**0.010**	**0.454**	**0.002**
Posterior WT	**0.596**	**0.041**	0.518	0.058	**0.405**	**0.011**	**0.424**	**0.031**	**0.432**	**0.006**	0.206	0.185
Relative WT	**0.579**	**0.048**	− 0.103	0.727	0.224	0.170	0.257	0.205	0.252	0.122	0.064	0.684
LV mass	**0.661**	**0.019**	**0.626**	**0.017**	**0.500**	**0.001**	0.351	0.079	0.431	0.006	**0.348**	**0.022**
LV index	0.415	0.180	0.458	0.099	**0.477**	**0.002**	**0.453**	**0.020**	**0.479**	**0.002**	0.224	0.149
Systolic function												
LV ejection fraction	− 0.097	0.765	0.123	0.675	− **0.358**	**0.025**	− 0.242	0.234	0.150	0.361	− 0.020	0.901
Fractional shortening	− 0.102	0.753	0.159	0.586	− **0.337**	**0.036**	− 0.245	0.227	0.168	0.308	− 0.009	0.955
s'-wave	− 0.405	0.191	− 0.217	0.457	0.077	0.643	− 0.036	0.863	− 0.220	0.179	0.023	0.882
Diastolic function												
Mitral valve inflow peak E	− 0.173	0.591	0.076	0.796	0.089	0.591	− 0.072	0.728	0.093	0.573	0.083	0.597
Mitral valve inflow peak A	− 0.394	0.205	0.174	0.553	− 0.071	0.669	− 0.121	0.556	− 0.011	0.948	0.054	0.733
E/A ratio	0.112	0.729	− 0.144	0.623	0.138	0.403	0.042	0.840	0.062	0.706	− 0.002	0.989
Mitral valve inflow Dt	0.504	0.094	− 0.004	0.990	0.055	0.740	− 0.210	0.302	− 0.249	0.126	0.195	0.211
eʹ-wave	− 0.555	0.061	0.145	0.621	0.027	0.873	0.161	0.433	− 0.193	0.240	− 0.120	0.442
aʹ-wave	− 0.298	0.346	− 0.213	0.465	− 0.255	0.117	− 0.090	0.663	− 0.180	0.272	0.136	0.384
eʹ/aʹ	− 0.317	0.315	0.165	0.572	0.200	0.222	0.195	0.339	− 0.004	0.981	− 0.164	0.293
E/e'	0.289	0.362	− 0.116	0.693	0.069	0.676	− 0.217	0.287	0.265	0.103	0.115	0.463

Relative wall thickness = (2 × posterior wall thickness)/LV end-diastolic diameter; bolded values indicate significant (p ≤ 0.05), *moderate-very large* correlation.

*LV* left ventricular, *WT* wall thickness, *Dt* deceleration time, *s'-wave* systolic mitral annulus velocity (average of septal and lateral measurements), *e'-wave* early diastolic myocardial relaxation (average of septal and lateral measurements), *a'-wave* active atrial contraction in late diastole (average of septal and lateral measurements).

### Structural parameters

Structural parameters were significantly (p < 0.001–0.011; ${\eta }_{p}^{2}$ = 0.08–0.63, *minimum-moderate*) different between groups. Post-hoc analyses revealed a significantly larger left atrium diameter in each athlete group (all p < 0.001 except long-distance runners, p = 0.014) compared to sedentary controls. Sedentary controls displayed a significantly smaller aortic root diameter than volleyball athletes (p = 0.03), as well as a significantly smaller aortic root index than football athletes (p = 0.002). Further, a significantly larger LV end-diastolic diameter was observed in long-distance runners (p = 0.030), volleyball athletes (p < 0.001), and football athletes (p < 0.001) compared to sedentary controls, in volleyball athletes compared to bodybuilders (p < 0.001) and powerlifters (p < 0.001), as well as in long-distance runners (p = 0.023) and football athletes (p < 0.001) compared to bodybuilders. Likewise, volleyball athletes (p = 0.006) and football athletes (p = 0.011) had a significantly larger LV end-systolic diameter than bodybuilders. In addition, ISWT, PWT, and RWT were significantly larger in bodybuilders and powerlifters (p < 0.001) compared to long-distance runners, volleyball athletes, football athletes, and sedentary controls. ISWT and PWT were also significantly larger in volleyball athletes (p = 0.001, p = 0.013) and football athletes (p = 0.012, p = 0.003) compared to sedentary controls. LV mass was significantly heavier in each athlete group (all p < 0.001 except long-distance runners, p = 0.007) compared to sedentary controls. Furthermore, LV mass was significantly heavier in powerlifters (p < 0.001) and bodybuilders (p = 0.009) compared to football athletes, as well as in powerlifters (p = 0.004) compared to long-distance runners. Likewise, LV mass index was significantly higher in each athlete group (all p < 0.001) compared to sedentary controls, as well as in long-distance runners (p = 0.007), volleyball athletes (p = 0.030), and football athletes (p < 0.001) compared to powerlifters.

Significant, *moderate*-*very large* correlations were found between corrected QTd and ISWT in long-distance runners (r = 0.712, p = 0.009), football athletes (r = 0.517, p = 0.001), powerlifters (r = 0.559, p = 0.003), bodybuilders (r = 0.406, p = 0.010), and sedentary controls (r = 0.454, p = 0.002). Similarly, significant, *moderate*-*large* correlations were found between corrected QTd and PWT in long-distance runners (r = 0.596, p = 0.041), football athletes (r = 0.405, p = 0.011), powerlifters (r = 0.424, p = 0.031), and bodybuilders (r = 0.432, p = 0.006). Significant, *moderate* correlations were found between corrected QTd and LV index in football athletes (r = 0.477, p = 0.002), powerlifters (r = 0.453, p = 0.020), and bodybuilders (r = 0.479, p = 0.002). Also, corrected QTd was significantly (*moderate*-*large*) correlated with RWT in long-distance runners (r = 0.579, p = 0.048), LV end-diastolic diameter in volleyball athletes (r = 0.564, p = 0.036), LV end-systolic diameter in football athletes (p = 0.010, r = 0.409), and aortic root diameter in sedentary control (p = 0.005, r = 0.419).

### Systolic function

LV ejection fraction and fractional shortening were significantly (p < 0.001; ${\eta }_{p}^{2}$ = 0.21–0.22, *minimum*) different between groups. A significantly higher LV ejection fraction was observed in long-distance runners (p < 0.001), volleyball athletes (p = 0.001), and football athletes (p = 0.014) compared to sedentary controls, in long-distance runners compared to powerlifters (p = 0.004) and bodybuilders (p < 0.001), as well as in volleyball athletes compared to bodybuilders (p = 0.011). Likewise, a significantly higher fractional shortening was observed in long-distance runners (p < 0.001), volleyball athletes (p < 0.001), and football athletes (p = 0.002) compared to sedentary controls, as well as in long-distance runners and volleyball athletes compared to powerlifters (p = 0.001 and p = 0.035) and bodybuilders (p < 0.001 and p = 0.007).

Significant inverse, *moderate* correlations were found between corrected QTd and echocardiographic parameters indicative of systolic function, including LV ejection fraction (r = − 0.358, p = 0.025) and fractional shortening (r = − 0.337, p = 0.036) only in football athletes.

### Diastolic function

Mitral valve inflow peak A, E/A ratio, and e'/a' were significantly (p = 0.002–0.006; ${\eta }_{p}^{2}$ = 0.09–0.11, *minimum*) different between groups. A significantly faster mitral valve inflow peak A (p = 0.006) was observed in bodybuilders compared to football athletes. Bodybuilders exhibited a significantly lower E/A ratio compared to long-distance runners (p = 0.016) and football athletes (p = 0.040). Furthermore, a significantly higher e'/a' was observed in volleyball athletes compared to football athletes (p = 0.029) and bodybuilders (p = 0.002), as well as in long-distance runners compared to sedentary controls (p < 0.001).

## Discussion

Our findings suggest QTd and LV remodeling in athletes are affected by the sport in which they compete. Advanced LV hypertrophy, predominantly concentric in nature, induced by pressure overload appears to be accompanied with an increased QTd in athletes predominantly completing static exercise (powerlifters and bodybuilders). On the other hand, mild LV hypertrophy induced by volume overload in long-distance runners, volleyball athletes, and football athletes appears to be accompanied with a reduced QTd. These hypertrophic responses appear to be physiological adaptations to the exercise load stimuli experienced by each group of athletes, without impairing systolic and diastolic function.

We observed longer RR-intervals in most athlete groups (long-distance runners, p = 0.15; volleyball athletes, football athletes, and powerlifters, p < 0.05) compared to sedentary controls. Our data align with meta-analytic evidence encompassing 298 participants who completed at least 4 weeks of aerobic exercise^28^ which found an exercise-induced bradycardia at rest resulting in a significantly (p < 0.001) longer RR-interval compared to sedentary controls. In this way, longer RR-intervals observed in long-distance runners, volleyball athletes, and football athletes were also accompanied by reduced QTd and corrected QTd compared to sedentary controls, powerlifters, and bodybuilders in our study. These findings contrast past observations reporting similar (adult male and female soccer athletes, runners, badminton athletes, basketball athletes, wrestlers, cyclists, handball athletes vs. sedentary controls, p = 0.7^13^; adult male basketball athletes, fencing athletes, gymnasts, judokas, swimming athletes, tennis athletes, volleyball athletes, and track and field athletes vs. non-athletes, p > 0.05^14^; middle-aged male distance runners vs. healthy controls, p = 0.6^18^; adult male long-distance runners vs. sedentary controls; p = 0.6^19^) or higher corrected QTd (male and female adolescent basketball athletes vs. untrained controls; p = 0.001^16^; male and female, adult middle-distance runners and football athletes vs. sedentary controls, p < 0.001^20^) in athletes from various sports compared to controls. Discrepancies in study findings may relate to several factors since exercise-induced adaptations produce varied cardiac remodeling in response to volume overload (during exercise involving isotonic muscle contractions) or pressure overload (during exercise involving isometric muscle contractions)^14,29^, as well as in male athletes compared to female athletes^14,29^, and in adults compared to adolescent or middle-aged athletes^29^. On the other hand, our findings agree with data reported in male athletes competing in highly dynamic sports who had a shorter corrected QTd compared to athletes predominantly undertaking static exercise (adolescent runners and football athletes vs. wrestlers and boxers: 50.8 ± 19.3 ms vs. 53.6 ± 17.2 ms, p = not reported^30^; adult basketball athletes, fencers, swimmers, tennis athletes, and volleyball athletes vs. gymnasts, judokas, and throwing and jumping athletes: 44.7 ± 11.9 ms vs. 50.1 ± 14.3 ms, p < 0.05^14^) and sedentary controls (adult endurance-trained athletes vs. less-trained controls involved in exercise < 2 × 30 min·week^−1^: 42.0 ± 13.0 ms vs. 51.0 ± 15.0 ms, p = 0.012^17^). Previous evidence suggests that the nature of the stress on the heart (volume or pressure overload) predominantly determines the phenotype^31^. Although physiological hypertrophy is initially induced as a compensatory response to pressure overload, with concentric growth of the ventricle, this type of hypertrophy may progresses into pathological LV hypertrophy characterized by preferential cardiomyocytes thickening as opposed to lengthening, with sarcomeres aligned in parallel^32,33^. Intermittent pressure overload activates signaling pathways that lead to pathological molecular, structural, and functional changes dissimilar to changes seen following endurance-based exercise (swimming or running)^31,33^. In this regard, cardiovascular events and deaths have been reported to occur in a higher (p < 0.05) proportion of hypertensive patients with concentric hypertrophy (events: 31%; deaths: 21%) compared to eccentric hypertrophy (events: 23%; deaths: 10%)^34^. Collectively, our findings indicate reduced QTd in long-distance runners, volleyball athletes, and football athletes, which reflects homogeneous ventricular repolarization in dynamic-trained athletes compared to the general population and athletes predominantly undertaking static exercise^35^.

Both groups of predominantly static-trained athletes (powerlifiters: 60.3 ± 7.3 ms and bodybuilders: 57.4 ± 5.7 ms) possessed significantly greater corrected QTd compared to sedentary controls (48.0 ± 8.2 ms). Our findings align with previous cross-sectional data demonstrating a significantly (p < 0.01) higher corrected QTd in athletes mainly undertaking static exercise (elite gymnasts: 61.7 ± 9.3 ms) compared to non-athletes (45.6 ± 10.9 ms)^14^. Together, these data parallel those observed in bodybuilders who were using large doses of androgenic anabolic steroids (range: 63–175 mg·day^−1^)^19^. In this previous research, bodybuilders exhibited longer corrected QTd (61.0 ± 12.0 ms vs. 52.0 ± 14.0 ms, p = 0.08) compared to sedentary controls^19^. While findings from our study should be interpreted cautiously due to participants self-reporting no anabolic steroid use, longer QTd in static-trained athletes compared to sedentary controls may relate to exercise-induced cardiac remodeling with increased ISWT^17^. In support of this assertion, existing evidence^36^ in hypertensive patients with LV hypertrophy suggests an increase in QTd occurs in patients with ISWT > 12 mm. Also, parallel increases in QTd and ISWT were further underpinned by correlation data for most athlete groups (long-distance runners, football athletes, powerlifters and bodybuilders) and the sedentary controls we examined. Advanced LV hypertrophy, as we observed in powerlifters and bodybuilders, is associated with reduced action potential amplitude, reduced membrane potential, shortened action potential duration, or electrical quiescence in cardiac cells^37^, which may underpin the increased QTd in these groups. In turn, a better understanding regarding the role of cardiac mass and ISWT in mediating QTd needs to be elucidated in future research. In addition, future studies are warranted to investigate the mechanisms underlying the associations between QTd with PWT and LV mass.

Our echocardiographic data indicative of structural parameters suggest different cardiac responses were apparent across groups, which is likely related to the stress imposed during the various forms of exercise they undertake. Given the different athlete groups predominantly completed dynamic (long-distance runners, football athletes, and volleyball athletes) or static exercises (powerlifters and bodybuilders), distinct load-specific patterns in LV hypertrophy emerged among them. Namely, normal (LV mass index < 115 g/m^2^ and RWT < 0.42) or eccentric LV hypertrophy (LV mass index > 115 g/m^2^ and RWT < 0.42) were observed in most long-distance runners (58% and 33%), volleyball athletes (50% and 50%), and football athletes (56% and 41%) likely due to the chronic volume overload they experienced throughout their exercise history. On the other hand, concentric LV hypertrophy (LV mass index > 115 g/m^2^ and RWT > 0.42) was observed in most powerlifters (58%) and bodybuilders (54%) likely due to the chronic pressure overload they experienced throughout their exercise history. Further, athletes participating in dynamic sports (long-distance runners, football athletes, and volleyball athletes) displayed ISWT (~ 11 mm) and LV PWT (~ 10 mm) within the normal range for the general population (IWST =  < 12 mm; LV PWT = 6–10 mm). In contrast, powerlifters and bodybuilders displayed ISWT (~ 12.5 mm) and LV PWT (~ 11.5 mm) exceeding the normal range for the general population. These structural parameters we observed in powerlifters and bodybuilders represent remodeling adaptations in response to training that fall within a 'grey zone' where extreme expressions of athlete’s heart and mild morphological forms of hypertrophic cardiomyopathy may overlap. Although these likely remodeling adaptations in powerlifters and bodybuilders mimic pathological hypertrophic cardiomyopathy, their LV end-diastolic diameters (~ 52–53 mm) were within clinically accepted partition values for the general population (45–55 mm), which is a useful and sensitive marker in distinguishing an athletic heart from hypertrophic cardiomyopathy diagnosis. Our results parallel to those reported in a previous meta-analysis^10^ including strength-trained athletes (weightlifters, powerlifters, bodybuilders, throwing athletes, and wrestlers: n = 544) who exhibited a significantly larger RWT (0.44 vs. 0.39, p = 0.006) and ISWT (11.8 mm vs. 10.5 mm, p = 0.005), as well as a trend toward a larger PWT (11.0 mm vs. 10.3 mm, p = 0.078) compared to long-distance runners (n = 413). Collectively, the observed structural differences across groups may be considered as sport-specific physiological adaptations, that are largely benign and related to the exercise histories of each group.

In addition to LV structural parameters, LV systolic indicators were also within clinically accepted reference ranges for the general population (ejection fraction > 53%^24^, fraction shortening 25–43%^38^, and s'-wave 7.2–12.9 cm/s^39^), further supporting adaptive (rather than maladaptive) physiological cardiac remodeling in athlete groups predominantly undertaking dynamic (long-distance runners, football athletes, and volleyball athletes) or static exercise (powerlifters and bodybuilders). Although we observed a significantly higher ejection fraction and fractional shortening at rest in long-distance runners compared to athletes mainly undertaking static exercise, as well as in athletes namely completing dynamic exercise compared to sedentary controls, effect size analyses revealed *minimal* ${(\eta }_{p}^{2}$ = 0.21–0.22) differences between comparison groups. Our data align with findings from previous meta-analytic evidence (n = 59 studies)^10^ showing comparable contractility between athletes predominantly undertaking dynamic exercise (long-distance runners: 68.8% [65.1–72.6%]), static exercise (weightlifters, powerlifters, bodybuilders, throwing athletes, and wrestlers: 66.3% [60.7–71.9%]), or mixed exercise regimes (cyclists and rowers: 66.1% [62.9–69.3%]), and sedentary controls (67.2% [64.5–69.8%]). In contrast to our findings, some evidence suggests that male athletes predominantly undertaking dynamic exercise demonstrate diminished ejection fractions at rest compared to sedentary controls (professional soccer athletes vs. untrained controls: 65 ± 4% vs. 70 ± 5%, p < 0.01^40^; professional cyclists vs. sedentary controls: 61.6 ± 6.4% vs. 65.3 ± 6.7%, p < 0.001^41^). An identified mechanism underlying the impaired LV contractility observed in endurance-trained athletes is LV cavity enlargement to assist in establishing adequate stroke volume to perfuse the body. In this regard, Abergel et al.^41^ reported a higher LV index (141 ± 21 g/m^2^) in world-class professional cyclists than we observed in long-distance runners (112 ± 11 g/m^2^), volleyball athletes (115 ± 14 g/m^2^), football athletes (112 ± 15 g/m^2^), powerlifters (128 ± 13 g/m^2^), and bodybuilders (120 ± 11 g/m^2^).

Left ventricular diastolic function is commonly assessed with E/A ratio and eʹ-wave to help identify the nature of hypertrophy as well as deceleration time to indicate LV compliance. Given the reference limits for normal diastolic function are 0.8–2.0 for E/A ratio^39^, > 0.12 m/s for e'-wave^23^, and 160–240 ms for deceleration time^39^, we observed normal diastolic function in all groups. Although differences were *minimal*, our data revealed E/A ratio was significantly higher in long-distance runners and football athletes compared to bodybuilders. Similarly, Utomi et al.^42^ reported an improved diastolic filling in male endurance-trained athletes compared to strength-trained athletes (E/A 2.0 [1.9–2.1] vs. 1.9 [1.7–2.0]). Compared to physiological cardiac hypertrophy, pathological hypertrophy is accompanied by disturbances in resting diastolic parameters. Specifically, in hypertensive patients increased LV mass and wall thickness is associated with diastolic filling abnormalities^43^. Comparable to concentric hypertrophy, it was found that eccentric LV hypertrophy produces diastolic filling pattern abnormalities in obese patients, characterized by diminished E/A ratio and prolonged deceleration time^11^. Nevertheless, our collective echocardiographic findings indicate cardiac variations between groups with different exercise histories occur primarily at a structural level rather than a functional level.

Some limitations of our investigation should be acknowledged when interpreting the findings. First, use of performance-enhancing drugs was self-reported among participants with no urine or serum measurements taken. Accordingly, drug screening is warranted to ensure athletes are not using banned substances, which may affect cardiac remodeling and/or QTd^19^. Second, our data are limited to males in selected sports. In turn, the provided results may not be indicative of female athletes and athletes competing in other sports (e.g., basketball, hockey, American football, handball, swimming, tennis) given cardiac remodeling and QTd have been shown to vary across sexes^14,29^ and load stimuli^14,29^. Third, although adult, national-level athletes with at least 3 years of training and competitive experience were recruited in our study, differences in the training programs undertaken across athletes may have contributed to the inter-individual variations in electrocardiographic and echocardiographic parameters. Fourth, in recruiting national-level participants across various sports, only 12 long-distance runners and 14 volleyball athlete participated in this study. Consequently, large cohort studies examining QTd and echocardiographic parameters in male and female athletes competing across a wider range of sports should be conducted in the future.

## Conclusion

Our findings suggest QTd and LV remodeling differs among athletes depending on the type of sport they compete in. Advanced LV hypertrophy, predominantly concentric in nature, was evident with an accompanying higher QTd in athletes predominantly undertaking static exercise (powerlifters and bodybuilders). On the other hand, LV hypertrophy toward the upper threshold of the normal reference range with an accompanying shorter QTd was apparent in athletes predominantly completing dynamic exercise (long-distance runners, volleyball athletes, and football athletes). Our collective data indicate that athletes competing in various sports may experience alterations in structural cardiac parameters that reflect physiological hypertrophy with normal systolic and diastolic function. Despite physiological hypertrophy, significantly longer QTd in powerlifters and bodybuilders indicate that sports practitioners predominantly prescribing static exercise should regularly perform electrocardiographic and echocardiographic screening to assist in capturing impaired ventricular repolarization among their athletes.

## Data Availability

The data that support the findings of this study are available upon request from the corresponding author.
